# Association of nutritional status and health-related quality of life in children with chronic kidney disease

**DOI:** 10.1007/s11136-019-02104-0

**Published:** 2019-01-12

**Authors:** Matthew Harmer, Stephen Wootton, Rodney Gilbert, Caroline Anderson

**Affiliations:** 1 Southampton Children’s Hospital, University Hospital Southampton NHS Foundation Trust, Tremona Road, Southampton, SO16 6YD UK; 2 University of Southampton, University Road, Southampton, SO17 1BJ UK; 3 Department of Nutrition and Dietetics, University Hospital Southampton NHS Foundation Trust, Tremona Road, Southampton, SO16 6YD UK; 4 NIHR Southampton Biomedical Research Centre–Nutrition, University Hospital Southampton NHS Foundation Trust, Southampton General Hospital, E-level, Tremona Road, Southampton, SO16 6YD UK

**Keywords:** Health-related quality of life, Children, Chronic kidney disease, Nutrition

## Abstract

**Purpose:**

Health-related quality of life (HRQoL) is an important, patient-centred measure. Although nutritional status is altered in children with CKD, the impact of nutritional status on HRQoL in this population has not been explored. The aims of this study are to report the HRQoL scores as assessed by the validated PedsQL™ questionnaire and to explore the relationship of HRQoL scores to markers of nutritional status. It will also examine the concordance between the scores of the child and their parent/carer.

**Methods:**

A single-centre, cross-sectional, observational study was performed exploring the markers of nutritional status (anthropometry—including presence of obesity, micronutrient status and appetite) and HRQoL and assessed by the PedsQL™ questionnaire in children aged 3–18 years with pre-dialysis, conservatively managed CKD.

**Results:**

A total of 46 children were recruited, with a mean age of 10.5 years. HRQoL scores were lower than in healthy controls throughout all domains. Lower scores were associated with short stature and poor appetite. Markers of obesity or micronutrient status were not associated with HRQoL scores.

**Discussion:**

Nutritional status impacts upon HRQoL. Further study is needed to evaluate how changing nutritional status may affect HRQoL in children with CKD, and this may be used to facilitate the development of patient-centred treatment goals and plans.

**Electronic supplementary material:**

The online version of this article (10.1007/s11136-019-02104-0) contains supplementary material, which is available to authorized users.

## Background

Chronic kidney disease (CKD) is an increasing problem in the UK and worldwide [1]. The disease and its management can have significant impacts upon the individual and their family, being associated with increased mortality and morbidity, and in children impaired growth [2].

Health-related quality of life (HRQoL) is an individual’s subjective perception of the impact of health status, including disease and treatment, on physical, psychological and social functioning [3]. Children with CKD, even those with disease on the milder end of the spectrum, have poorer HRQoL [4–7]. The physical and psychological impact of both the disease process itself and its management make CKD a particularly challenging condition with pervasive symptoms of nausea and lethargy, multiple clinic attendances and investigations, strict dietary modification, multiple medications (and their frequent adjustments), changes in physical health due to complications of CKD and psychological problems such as altered body-image, anxiety and depression.

HRQoL is not only determined by disease severity. In children with CKD, Gerson et al found that renal function did not correlate with HRQoL [5]. Factors other than the disease itself contribute to HRQoL in those children with disease [8], and the authors hypothesise that nutritional status impacts HRQoL. If such an association exists, then HRQoL assessment may off a measure of how nutritional status impacts the child in a holistic, patient-centred way.

Malnutrition is common in CKD [9] and is associated with poorer outcomes [10]. It has been traditionally defined as poor nutritional status resulting from inadequate intake, but in CKD malnutrition is multifactorial in origin, with suppressed appetite, catabolism and chronic inflammation, altered dietary intake from dietetic therapy and nutrient losses through dialysis all contributing. In addition to under-nutrition, over-nutrition and obesity are becoming increasingly prevalent in CKD populations across the UK and Europe [11] and more prevalent than canonical malnutrition state of low anthropometric scores and lean tissue loss in both the conservatively managed [12] and dialysis/post-transplant populations [11]. Obesity has been reported to be an independent risk factor for development of renal damage and end-stage renal failure [13, 14].

In cohorts of children with other disease states, poor nutritional status [15] and obesity [16] have been associated with poorer HRQoL, but this has not been explored in paediatric CKD. Micronutrient status has been associated with HRQoL [17], including selenium [18] with supplementation of selenium demonstrating improved scores [19–21], although there are contradictory findings [22].

Despite the recognition that HRQoL is important, there are limited data in this disease group with existing literature focusing upon end-stage disease and those following renal transplantation. Of the studies that examine CKD stages 2–4 in the paediatric population [5, 6, 23–26], only one [25] examines nutritional status (evaluating the effect of short stature). There is therefore, a need to examine the effect of nutritional status, including obesity on HRQoL, and any association of HRQoL on future nutritional status.

Describing the subjective assessment of HRQoL of both the child and their caregiver is important as healthcare providers’ focus is that of the child, but some children may be unable to describe their experiences, and caregivers make choices (including medical) dependent upon their assessment of the child’s HRQoL. Discordance between self-reported and parent-proxy HRQoL scores has been reported previously in those with dialysis and post-renal transplantation [27–29], but not assessed in those with less severe disease.

The aims of this study are to report the HRQoL scores as assessed by the validated PedsQL™ questionnaire and to explore the relationship of HRQoL scores to markers of nutritional status. It will also examine concordance between the scores of the child and their parent/carer.

## Methods

A cross-sectional, observational study was performed to determine the HRQoL of a cohort of children with pre-dialysis, conservatively managed CKD and its relationship to markers of nutritional status.

### Participants

Children aged between 3 and 18 years with conservatively managed CKD (stages 2–5 as defined by Kidney Disease: Improving Global Outcomes (KDIGO) [30] [Pre-end-stage disease—glomerular filtration rate between 15 and 90 ml/min/1.73 m^2^], not receiving dialysis and never received a renal transplant) under care of the paediatric nephrology team at Southampton Children’s Hospital were identified using electronic notes, approached via a letter of invitation and subsequently assessed at routine clinic appointments. Both the child and a parent/caregiver were requested to independently complete the respective questionnaire.

### Measures

Basic clinical and anthropometric data were collected, including height, weight, waist circumference, mid-upper arm circumference (MUAC) and standard deviation score (SDS), body mass index (BMI) and waist circumference-to-height ratio (WHtR). Appetite was assessed by a simple Likert scale by the child: Very poor-1, Poor-2, Good-3, Very good-4. Definitions of obesity were BMI SDS > 2; MUAC SDS > 2 or > 25 cm [31] and a WHtR > 0.5 [32]. Short stature was defined as a height SDS < − 2. Blood for analysis of markers of the micronutrients copper, selenium, zinc and manganese were collected and analysed through clinical pathology.

Further anthropometric and clinical data were collected via the electronic clinical record at 6 months and 12 months subsequent to initial assessment, and change in weight SDS, BMI SDS, height SDS and estimated glomerular filtration rate (eGFR) calculated.

The HRQoL was assessed using the PedsQL™ tool [33]. This is a series of questions posed to the child and the parent/caregiver that assess physical, emotional, social and schooling aspects of the child’s life.

The PedsQL™ tool was developed to measure HRQoL in children and has been validated for a number of chronic health conditions [34–36]. It was highlighted in a systematic review of tools to assess HRQoL as one of the more thoroughly developed measures and has been validated for a wide age range of children [37]. Although originally developed and validated for a USA population, it has been validated for a UK population of both healthy children and those with chronic conditions (asthma, diabetes and inflammatory bowel disease) [38].

The PedsQL™ [33] comprises two questionnaires: one for the child and one for a parent/caregiver. They ask a series of questions that assesses four domains: physical, emotional, social and schooling aspects of the child’s life. It is scored as individual domains and as a total score (all four domains), and is expressed as a percentage of maximum score. The tool is available in age-appropriate versions: toddler (2–4 years), young child (5–7 years), older child (8–12 years) and teenager (13–18 years). There is no child (self-rater) questionnaire for those aged 2–4 years.

As a marker of deprivation, Income Deprivation Affecting Children Index (IDACI) scores were calculated using participants’ postcodes. United Kingdom Department of Communities and Local Government data (2015) via the online platform provide a selection of official statistics and data outputs on a variety of themes related to the department of Communities and Local Government (http://opendatacommunities.org).

### Statistical analysis

Data were analysed using SPSS version 20 for Windows (SPSS Inc., Chicago, Illinois, United States of America). Statistical significance was defined a *p* value of less than 0.05. Descriptive statistical analysis was performed, including distribution of gender and age group (as defined by the PedsQL™ questionnaire).

Independent t-tests were performed to compare: the total questionnaire scores with both healthy control data from Varni et al’s cohort [33]. Concordance between self-reported and parent-proxy questionnaire scores was compared using a paired t-test.

Correlation was explored between HRQoL scores and markers of nutritional status by examination of scatter-plot, and if appropriate, calculation of correlation coefficient values (Spearman’s rho). Internal consistency between the domains of the questionnaires was tested using Cronbach’s alpha.

### Ethical approval

The study was approved by a Health Research Authority South East—Surrey Research Ethics Committee (REC Reference: 16/LO/0041). Informed consent was obtained from parents/carers with parental responsibility. Informed assent was additionally obtained from those children/young people as participants in the study if age appropriate.

## Results

A total of 46 children with pre-dialysis, conservatively managed CKD were recruited with additional seven families refusing to take part in the study. These seven families did not represent a particular sub-group (a mix of degree of renal impairment levels, age and gender). The cohort had a mean age of 10.50 ± 4.19 years. Eighteen (39.1%) were female. The demographic and anthropometric details of the cohort are given in Table 1. There were no significant differences between boys and girls; including age, time since diagnosis, eGFR, height SDS, weight SDS, BMI SDS, WHtR and appetite.

**Table 1:** Demographic and anthropometric data of the cohort paediatric pre-dialysis, conservatively managed CKD

Variable	Averages
Age (years)	10.50 (SD ± 4.19)
Gender	Male = 28 (60.87%), Female = 18 (39.13%)
eGFR (ml/min/1.73 m^2^)	57.35 (SD ± 23.95)
Number of medications	3.89 (SD ± 2.54)
Time since diagnosis (months)	93.27 (SD ± 55.34)
Height SDS^a^	− 0.65 (IQR = 2.03)
Weight SDS	− 0.43 (SD ± 1.81)
BMI SDS	0.32 (SD ± 1.41)
MUAC SDS	0.52 (SD ± 0.92)
HtWR	0.49 (SD ± 0.07)

*CKD* chronic kidney disease, *eGFR* estimated glomerular filtration rate, *MUAC* mid-upper arm circumference, *SD* standard deviation, *SDS* standard deviation score, *WHtR* waist circumference-to-height ratio

^a^Median and interquartile range

### Anthropometry

Mean values (with standard deviations) of height SDS, weight SDS and BMI SDS for the cohort were − 1.03 (± 1.51), -0.43 (± 1.81) and 0.32 (± 1.41), respectively. The majority of children were within normal growth limits (± 2SD). A greater number of children were obese as defined by WHtR than by Wt SDS or BMI SDS definitions. Eight children (17.4%) had weight SDS < − 2 (malnourished by ICD-10 definition), three children (6.5%) had weight SDS > 2. One child (2.2%) was underweight for height (BMI SDS < − 2), six children (13.0%) were overweight for height (BMI SDS > 2). Twelve children (26.1%) were short-for-age (height SDS < − 2), one child (2.2%) was tall-for-age (height SDS > 2). Twenty children (43.5%) had a waist circumference-to-height ratio greater than 0.5.

### HRQoL scores

All participants enrolled in the study fully completed the appropriate questionnaires. HRQoL scores are presented in Table 2. HRQoL scores were lower in all domains for both the self-rated and parent-proxy components of the PedsQL™ tool than healthy control populations (Varni et al). There was good concordance between the self-rater and parent-proxy scores (*t* test: *n* = 38, *t*(37) = 0.281, *p* = 0.780). Details of sub-group analysis for different ages and PedsQL™ domains are available in the *supplementary material*. There was a high level of internal consistency between domains of the questionnaires with a Cronbach’s alpha of 0.810 (child self-rater) and 0.811 (parent-proxy).

**Table 2:** PedsQLTM scores for the cohort compared to healthy controls

Children with CKD at SCH (self-rater)					Healthy control data (Varni et al)				
Physical	Emotional	Social	School	Total	Physical	Emotional	Social	School	Total
65.10 (22.57)	34.38 (24.65)	65.48 (23.98)	58.88 (16.91)	64.22 (18.00)	86.86 (13.88)*	78.21 (18.64)*	84.04 (17.43)*	79.92 (16.93)*	82.87 (13.16)*

Children with CKD at SCH (parent-proxy)					Healthy control data (Varni et al)				
Physical	Emotional	Social	School	Total	Physical	Emotional	Social	School	Total
65.27 (26.27)	61.47 (22.53)	70.52 (22.80)	63.48 (19.68)	64.64 (19.13)	83.26 (19.98)*	80.28 (16.99)*	82.15 (20.08)*	76.91 (20.16)*	81.34 (15.92)*

Note that there is no self-rated score for those aged < 5 years

*Significant difference for *t* test *p* < 0.0005

### HRQoL and renal function

Although HRQoL scores correlated with renal function (eGFR) for the child(self-rater) scores (Pearson’s coefficient = − 0.362, *p* = 0.026), this was not the case for the parent-proxy scores (Pearson’s coefficient = − 0.133, *p* = 0.377). Although there is correlation of self-rated PedsQL™ scores, there were no difference in PedsQLTM scores for either self-rater or parent-proxy components between CKD stages (self-rater: *F* = 2.284, df = 5, *p* = 0.07; parent-proxy: *F* = 1.117, df = 5, *p* = 0.367). Degree of proteinuria (urinary protein–creatinine ratio, uPCR) did not correlate with HRQoL scores, including individual domain scores (total score (child) Spearman’s rho = 0.185, *p* = 0.870; total score (parent-proxy) Spearman’s rho = − 0.05, *p* = 0.755).

### HRQoL and anthropometry (markers of growth and obesity)

Those who were stunted (Height SDS < − 2) had lower HRQoL scores than those who were not for both the child (*t*(15.0394) = − 3.4356; *p* = 0.0037) and parent questionnaires (*t*(22.87220) = − 4.10670; *p* = 0.0004). Those with obesity (as defined by BMI SDS, MUAC and waist circumference-to-height ratio) did not demonstrate different scores from non-obese patients. Children whose appetite was described as either “good” or “very good” had better scores than those with appetites described as “poor” or “very poor” (child—*t*(36) = 2.851; *p* = 0.007 and parent—*t*(44) = 2.910; *p* = 0.006).

### HRQoL and micronutrient status

Correlations were explored between the plasma levels of copper, selenium, zinc and whole blood manganese. None of these measures correlated with HRQoL scores with *p* values between 0.061 and 0.731 (analysis available as Supplementary Material).

### Multiple linear regression analysis

Variables that demonstrated correlation were used to perform multiple linear regression analysis.

#### Child (self-rater) HRQoL

On multiple linear regression for correlated variables (eGFR and Ht SDS), these two variables statistically significantly predicted child self-assessed HRQoL *F*(2,35) = 12.436, *p* < 0.0005, *R*^2^ = 0.415. Both Ht SDS and eGFR added statistically and significantly to the prediction of child-assessed HRQoL (Ht SDS *p* < 0.0005, eGFR *p* = 0.008).

#### Parent-proxy HRQoL

On multiple linear regression for positively correlated variables (Ht SDS and IDACI), these two variables statistically significantly predicted parent-proxy HRQoL *F*(2,42) = 11.695, *p* < 0.0005, *R*^2^ = 0.358. Both Ht SDS and IDACI added statistically significantly to the prediction of child-assessed HRQoL (Ht SDS *p* = 0.001, IDACI *p* = 0.005).

## Discussion

These are the first data of HRQoL as assessed by the PedsQL™ 4.0 in children with pre-dialysis, conservatively managed CKD within the UK, and they explore the association of nutritional status with HRQoL. The aim of this study was to report PedsQL™ scores in a UK population of pre-dialysis, conservatively managed CKD and explore the association of nutritional status on them. We hypothesised that disease severity, obesity and short stature would demonstrate association, but of the nutritional status measures evaluated, poor appetite and short stature demonstrated a strong association with lower HRQoL scores.

These data show that HRQoL was significantly lower in this population of pre-dialysis conservatively managed CKD children than healthy control data, with mean score differences greater than the minimal clinically important difference (MCID) score of 4.4 (child self-assessed questionnaire) and 4.5 (parent-proxy questionnaire) [33]. Existing literature is limited and has focused on those with the most severe disease—those with end-stage disease (eGFR < 15 ml/min/1,73 m^2^, in receipt of including dialysis, or who have undergone renal transplantation). On the whole, these report that HRQoL is lower in children with CKD compared to healthy children, but with some conflicting results [6, 23–25, 28, 39–46]. Such conflict may be explained by the heterogeneity of the tools used, but other factors may also be contributory.

There was no difference in scores according to age or gender in this cohort, although previous studies have shown lower scores in older children/young people [46] and in girls [45, 46]. Time since diagnosis did not correlate with HRQoL scores. This may be explained by the heterogeneity of the cohort, with a potential mix of those whose HRQoL worsens with time, and those that have altered “new normal”—a phenomenon described as “response shift” [47].

### HRQoL concordance between child (self-rater) and parent-proxy scores

There was significant correlation between child (self-rater) and parent-proxy scores, with no significant differences either in total scores or individual domains within any age groups. Discordance between self-reported and parent-proxy scores has been reported previously in those with dialysis and post-renal transplantation [27–29] with other literature showing greater psychosocial score discordance in those approaching adulthood [25, 48]. A degree of disagreement is not surprising as the child’s perception of quality of life will be different, at least in part due to parental expectations for their child based on their own life experiences; experiences that the child would not necessarily draw comparison with. This potential lack of comparison on the child’s part is one reason why HRQoL should be assessed by children and parents/caregivers in order not to over-score those that ‘don’t know any difference’. Previous studies that suggest that discordance between self-reported and parent-proxy questionnaires increases as the child’s age increases [25]. This is true not only for teenagers approaching adulthood, but demonstrated by Razzcuk et al, also or younger children [49]. The reason for these data not reflecting differences may be because of the relatively small numbers within the cohort, although the difference between child (self-rater) and parent-proxy scores were close to statistical significance in the emotional domain (*p* = 0.054).

### HRQoL and disease severity

Child (self-rater) HRQoL scores correlated with eGFR, but parent-proxy score did not, and no correlation was found between scores and degree of proteinuria. As the renal impairment would have greatest impact upon the individual, then other modifying factors on the caregiver may explain the lack of correlation observed in the parent-proxy scores. As proteinuria may be modified through medical intervention (such as the use of angiotensin II-converting enzyme inhibitors), proteinuria is not a complete marker of disease severity.

Although eGFR did correlate with child (self-rater) HRQoL scores in our cohort, Gerson et al found that eGFR did not correlate with HRQoL [5]. A lack of correlation may be explained by the fact that eGFR is not a true reflection of disease severity as it is too simplistic a marker. Furthermore, it does not take into consideration co-morbidities, social factors or other contributors to HRQoL.

### HRQoL and growth

As previously reported in other disease groups [25], stunted children reported lower HRQoL scores than their non-stunted counterparts, and it was the strongest influence on multiple regression analysis. The reasons for this may be twofold. Firstly, linear growth is a summation of events, is affected by myriad factors and is a proxy measure for everything detrimental that has happened to the child. Secondly, short stature may have psychological impact on the child, as both a visual reflection of a child’s self-perceived health, and a noticeable difference between themselves and their peers.

### HRQoL and obesity

Despite previous reports from other cohorts of children [50], these data do not demonstrate a lower HRQoL in those with markers of obesity (BMI SDS, waist circumference-to-height ratio). The reason for this may be a shift of the perceived normal body shape and “accepted” adiposity in children. Rates of obesity in the UK population are now 14% of children aged 2–15 years (BMI > 95th percentile) [51], and compare similarly to the percentage of those obese in this cohort (BMI > 2 SDS). Therefore, despite children being obese, if their day-to-day functioning is not negatively impacted by this, then such children do not stand out from their peers, and so are perceived as “normal”, or even healthy—both obese children and their parents’ ability to recognise themselves as too heavy is only 26% and ≤ 48%, respectively [51].

### HRQoL and appetite

HRQoL scores were significantly higher in those with good appetite versus poor appetite. As appetite is a complex function of many factors in which general health and well-being impact upon, this is not surprising. In addition, eating is an important social setting—on which much of family life is structured around. Alteration of feeding habits, including through eating less and a focus of the medicalisation of the normal social activity (advice to eat certain foods, avoid certain foods and to encourage oral intake in order to increase calorie intake, for example), may have an impact on how they view themselves compared to others, and hence their perceived HRQoL.

### HRQoL and micronutrient status

Biomarkers of micronutrient status did not correlate with HRQoL scores. These measurements are prone to many factors, and have a high degree of buffering within the normal range; it is only at the extremes of nutrition that these values become abnormal. In addition, levels may be affected by other factors such as underlying inflammatory state.

### Limitations of study

There are several limitations to this study. Firstly, the cohort although larger than other reported cohorts is only 46 children. Additionally, the study only measured HRQoL at a single time-point meaning that we were unable to examine for changing scores with time (including during changes in nutritional status and treatment). Nutritional status is a complex, multi-faceted concept and this study only examined a selection of variables. Each of these measures has its own limitations (for example, BMI not truly reflecting body composition). Not all variables that may have an impact on HRQoL, such as markers of resilience and patient efficacy, were explored.

### Future directions

Further exploration of factors that could be influencing HRQoL, especially those that are modifiable, is needed with the aim to improve the HRQoL of our patients. It is not unreasonable to predict that the nutritional changes, including dietary restriction found in CKD and changes of body composition, may have a detrimental effect upon HRQoL. Therefore, further studies analysing the impact would be valuable in order to optimise management strategies to improve HRQoL. A larger multi-centre cohort would allow for exploration of different aetiologies and the effect this may have on HRQoL in addition to minimising type 2 errors. Longitudinal studies of HRQoL in this disease group are also needed. Although there are cross-sectional data comparing treatment modalities, it would be useful to know how an individual’s HRQoL changes with time, with the changing disease severity, changing treatment modality, changing nutritional status, transitioning from paediatric to adult services. This would facilitate the exploration of the ways of different management strategies, including nutritional intervention influence HRQoL.

## Concluding remarks

HRQoL is lower in pre-dialysis, conservatively managed paediatric CKD patients than healthy control data. Examination of multiple nutritional variables revealed that nutritional status is associated with HRQoL. Other significant variables were eGFR for child (self-rater) scores and level of deprivation for parent-proxy scores.

HRQoL is a measure of an overall status, and the perception of an individual’s own health status is an important factor that healthcare providers should be aiming to improve. As healthcare services become more patient-centred, the measures by which they are evaluated must include patient-centred measures and by assessing HRQoL using a formalised tool, healthcare processes can be evaluated. HRQoL tools also have the potential to be used as screening tools or to identify areas to be explored during outpatient consultation for a more holistic consultation. For these reasons, HRQoL assessment should be considered for introduction into routine clinical care for ongoing holistic care of children with chronic illnesses, including CKD, and to facilitate the acquisition of longitudinal data regarding the impact of changes in nutritional status and therapy.

## Electronic supplementary material

Below is the link to the electronic supplementary material.

Supplementary material 1 (DOCX 20 KB)

## References

[1] Harambat J, van Stralen KJ, Kim JJ, Tizard EJ (2012). Epidemiology of chronic kidney disease in children. Pediatric Nephrology (Berlin, Germany).

[2] Greenbaum LA, Warady BA, Furth SL (2009). Current advances in chronic kidney disease in children: Growth, cardiovascular, and neurocognitive risk factors. Seminars in Nephrology.

[3] Leidy NK, Revicki DA, Geneste B (1999). Recommendations for evaluating the validity of quality of life claims for labeling and promotion. Value in Health: The Journal of the International Society for Pharmacoeconomics and Outcomes Research.

[4] Rees L (2009). Long-term outcome after renal transplantation in childhood. Pediatric Nephrology (Berlin, Germany).

[5] Gerson AC, Wentz A, Abraham AG, Mendley SR, Hooper SR, Butler RW, Gipson DS, Lande MB, Shinnar S, Moxey-Mims MM, Warady BA, Furth SL (2010). Health-related quality of life of children with mild to moderate chronic kidney disease. Pediatrics.

[6] McKenna AM, Keating LE, Vigneux A, Stevens S, Williams A, Geary DF (2006). Quality of life in children with chronic kidney disease-patient and caregiver assessments. Nephrology, dialysis, transplantation: Official publication of the European Dialysis and Transplant Association -. European Renal Association.

[7] Moreira JM, Bouissou Morais Soares CM, Teixeira AL, Simoes ESAC, Kummer AM (2015). Anxiety, depression, resilience and quality of life in children and adolescents with pre-dialysis chronic kidney disease. Pediatric Nephrology (Berlin, Germany).

[8] Stone MB, Botto LD, Feldkamp ML, Smith KR, Roling L, Yamashiro D, Alder SC (2010). Improving quality of life of children with oral clefts: Perspectives of parents. The Journal of Craniofacial Surgery.

[9] Fouque D, Kalantar-Zadeh K, Kopple J, Cano N, Chauveau P, Cuppari L, Franch H, Guarnieri G, Ikizler TA, Kaysen G, Lindholm B, Massy Z, Mitch W, Pineda E, Stenvinkel P, Trevino-Becerra A, Wanner C (2008). A proposed nomenclature and diagnostic criteria for protein-energy wasting in acute and chronic kidney disease. Kidney International.

[10] Wong CS, Gipson DS, Gillen DL, Emerson S, Koepsell T, Sherrard DJ, Watkins SL, Stehman-Breen C (2000). Anthropometric measures and risk of death in children with end-stage renal disease. American Journal of Kidney Diseases: The Official Journal of the National Kidney Foundation.

[11] Bonthuis M, van Stralen KJ, Verrina E, Groothoff JW, Alonso Melgar A, Edefonti A, Fischbach M, Mendes P, Molchanova EA, Paripovic D, Peco-Antic A, Printza N, Rees L, Rubik J, Stefanidis CJ, Sinha MD, Zagozdzon I, Jager KJ, Schaefer F (2013). Underweight, overweight and obesity in paediatric dialysis and renal transplant patients. Nephrology, dialysis, transplantation: Official publication of the European Dialysis and Transplant Association. European Renal Association.

[12] Rodig, N. M., McDermott, K. C., Schneider, M. F., Hotchkiss, H. M., Yadin, O., Seikaly, M. G., Furth, S. L., & Warady, B. A. (2014) Growth in children with chronic kidney disease: A report from the Chronic Kidney Disease in Children Study. *Pediatric Nephrology (Berlin, Germany), 29*, 1987–1995. 10.1007/s00467-014-2812-9 PMC4470271 24728472

[13] Hsu CY, McCulloch CE, Iribarren C, Darbinian J, Go AS (2006). Body mass index and risk for end-stage renal disease. Annals of Internal Medicine.

[14] Ribstein, J., du Cailar, G., & Mimran, A. (1995) Combined renal effects of overweight and hypertension. *Hypertension (Dallas, Tex: 1979), 26*, 610–615. 10.1161/01.hyp.26.4.610 7558220

[15] Brinksma A, Sanderman R, Roodbol PF, Sulkers E, Burgerhof JG, de Bont ES, Tissing WJ (2015). Malnutrition is associated with worse health-related quality of life in children with cancer. Supportive Care in Cancer: Official Journal of the Multinational Association of Supportive Care in Cancer.

[16] Buttitta M, Iliescu C, Rousseau A, Guerrien A (2014). Quality of life in overweight and obese children and adolescents: A literature review. Quality of Life Research: An International Journal Of Quality of Life Aspects of Treatment, Care and Rehabilitation.

[17] Duran Aguero S, Gonzalez Canete N, Pena D’Ardaillon F, Candia Johns P (2015). [Association of intake macro and micronutrients with life quality of life in elderly]. Nutricion Hospitalaria.

[18] Gonzalez S, Huerta JM, Fernandez S, Patterson AM, Lasheras C (2007). Life-quality indicators in elderly people are influenced by selenium status. Aging Clinical and Experimental Research.

[19] Witte KK, Nikitin NP, Parker AC, von Haehling S, Volk HD, Anker SD, Clark AL, Cleland JG (2005). The effect of micronutrient supplementation on quality-of-life and left ventricular function in elderly patients with chronic heart failure. European Heart Journal.

[20] Oh TR, Kim CS, Bae EH, Ma SK, Han SH, Sung SA, Lee K, Oh KH, Ahn C, Kim SW (2017). Association between vitamin D deficiency and health-related quality of life in patients with chronic kidney disease from the KNOW-CKD study. PloS ONE.

[21] Johansson P, Dahlstrom O, Dahlstrom U, Alehagen U (2015). Improved health-related quality of life, and more days out of hospital with supplementation with selenium and coenzyme Q10 combined. results from a double blind, placebo-controlled prospective study. The Journal of Nutrition, Health & Aging.

[22] Rayman M, Thompson A, Warren-Perry M, Galassini R, Catterick J, Hall E, Lawrence D, Bliss J (2006). Impact of selenium on mood and quality of life: A randomized, controlled trial. Biological psychiatry.

[23] Reynolds JM, Garralda ME, Jameson RA, Postlethwaite RJ (1988). How parents and families cope with chronic renal failure. Archives of Disease in Childhood.

[24] Heath J, Mackinlay D, Watson AR, Hames A, Wirz L, Scott S, Klewchuk E, Milford D, McHugh K (2011). Self-reported quality of life in children and young people with chronic kidney disease. Pediatric Nephrology (Berlin, Germany).

[25] Al-Uzri A, Matheson M, Gipson DS, Mendley SR, Hooper SR, Yadin O, Rozansky DJ, Moxey-Mims M, Furth SL, Warady BA, Gerson AC (2013). The impact of short stature on health-related quality of life in children with chronic kidney disease. The Journal of Pediatrics.

[26] Teixeira CG, Duarte Mdo C, Prado CM, Albuquerque EC, Andrade LB (2014). Impact of chronic kidney disease on quality of life, lung function, and functional capacity. Jornal de pediatria.

[27] Anthony SJ, Hebert D, Todd L, Korus M, Langlois V, Pool R, Robinson LA, Williams A, Pollock-BarZiv SM (2010). Child and parental perspectives of multidimensional quality of life outcomes after kidney transplantation. Pediatric Transplantation.

[28] Sundaram SS, Landgraf JM, Neighbors K, Cohn RA, Alonso EM (2007). Adolescent health-related quality of life following liver and kidney transplantation. American Journal of Transplantation: Official Journal of the American Society of Transplantation and the American Society of Transplant Surgeons.

[29] Varni JW, Limbers CA, Burwinkle TM (2007). Impaired health-related quality of life in children and adolescents with chronic conditions: A comparative analysis of 10 disease clusters and 33 disease categories/severities utilizing the PedsQL 4.0 Generic Core Scales. Health and Quality of Life Outcomes.

[30] Group KDIGOKCW (2013). KDIGO 2012 clinical practice guideline for the evaluation and management of chronic kidney disease. Kidney International.

[31] Chaput, J. P., Katzmarzyk, P. T., Barnes, J. D., Fogelholm, M., Hu, G., Kuriyan, R., Kurpad, A., Lambert, E. V., Maher, C., Maia, J., Matsudo, V., Olds, T., Onywera, V., Sarmiento, O. L., Standage, M., Tudor-Locke, C., Zhao, P., & Tremblay, M. S. (2016) Mid-upper arm circumference as a screening tool for identifying children with obesity: A 12-country study. *Pediatric Obesity*. 10.1111/ijpo.12162 27238202

[32] Ashwell M, Lejeune S, McPherson K (1996). Ratio of waist circumference to height may be better indicator of need for weight management. BMJ.

[33] Varni JW, Burwinkle TM, Seid M, Skarr D (2003). The PedsQL 4.0 as a pediatric population health measure: Feasibility, reliability, and validity. Ambulatory Pediatrics: The Official Journal of the Ambulatory Pediatric Association.

[34] Varni JW, Burwinkle TM, Jacobs JR, Gottschalk M, Kaufman F, Jones KL (2003). The PedsQL in type 1 and type 2 diabetes: Reliability and validity of the pediatric quality of life inventory generic core scales and type 1 diabetes module. Diabetes Care.

[35] Varni JW, Burwinkle TM, Katz ER, Meeske K, Dickinson P (2002). The PedsQL in pediatric cancer: Reliability and validity of the pediatric quality of life inventory generic core scales, multidimensional fatigue scale, and cancer module. Cancer.

[36] Chan KS, Mangione-Smith R, Burwinkle TM, Rosen M, Varni JW (2005). The PedsQL: Reliability and validity of the short-form generic core scales and Asthma Module. Medical Care.

[37] Eiser C, Morse R (2001). Quality-of-life measures in chronic diseases of childhood. Health Technology Assessment (Winchester, England).

[38] Upton P, Eiser C, Cheung I, Hutchings HA, Jenney M, Maddocks A, Russell IT, Williams JG (2005). Measurement properties of the UK-English version of the Pediatric Quality of Life Inventory 4.0 (PedsQL) generic core scales. Health and Quality of Life Outcomes.

[39] Diseth TH, Tangeraas T, Reinfjell T, Bjerre A (2011). Kidney transplantation in childhood: Mental health and quality of life of children and caregivers. Pediatric Nephrology (Berlin, Germany).

[40] Qvist E, Narhi V, Apajasalo M, Ronnholm K, Jalanko H, Almqvist F, Holmberg C (2004). Psychosocial adjustment and quality of life after renal transplantation in early childhood. Pediatric Transplantation.

[41] Goldstein SL, Graham N, Burwinkle T, Warady B, Farrah R, Varni JW (2006). Health-related quality of life in pediatric patients with ESRD. Pediatric Nephrology (Berlin, Germany).

[42] Eijsermans RM, Creemers DG, Helders PJ, Schroder CH (2004). Motor performance, exercise tolerance, and health-related quality of life in children on dialysis. Pediatric Nephrology (Berlin, Germany).

[43] Hamiwka LA, Cantell M, Crawford S, Clark CG (2009). Physical activity and health related quality of life in children following kidney transplantation. Pediatric Transplantation.

[44] Falger J, Landolt MA, Latal B, Ruth EM, Neuhaus TJ, Laube GF (2008). Outcome after renal transplantation. Part II: Quality of life and psychosocial adjustment. Pediatric Nephrology (Berlin, Germany).

[45] Neul SK, Minard CG, Currier H, Goldstein SL (2013). Health-related quality of life functioning over a 2-year period in children with end-stage renal disease. Pediatric Nephrology (Berlin, Germany).

[46] Marciano RC, Bouissou Soares CM, Diniz JSS, Lima EM, Silva JMP, Canhestro MR, Gazzinelli A, Melo CCD, Dias CS, Correa H, Oliveira EA (2011). Pediatric Nephrology.

[47] Sprangers MA, Schwartz CE (1999). Integrating response shift into health-related quality of life research: A theoretical model. Social Science & Medicine.

[48] Lopes M, Ferraro A, Koch VH (2014). Health-related quality of life of children and adolescents with CKD stages 4–5 and their caregivers. Pediatric Nephrology (Berlin, Germany).

[49] Razzouk BI, Hord JD, Hockenberry M, Hinds PS, Feusner J, Williams D, Rackoff WR (2006). Double-blind, placebo-controlled study of quality of life, hematologic end points, and safety of weekly epoetin alfa in children with cancer receiving myelosuppressive chemotherapy. Journal of Clinical Oncology: Official Journal of the American Society of Clinical Oncology.

[50] Schwimmer JB, Burwinkle TM, Varni JW (2003). Health-related quality of life of severely obese children and adolescents. JAMA.

[51] Conolly (2016) Health Survey for England 2015: Children’s BMI, overweight and obesity. Health and Social Care Information Centre.

