# Supplementary material for: Association of nutritional status and health-related quality of life in children with chronic kidney disease
Source: Qual Life Res. 2019 Jan 12;28(6):1565–73. doi: 10.1007/s11136-019-02104-0 (PMC6522445; doi:10.1007/s11136-019-02104-0)
Supplement: Supplementary file 1 — Supplementary material 1 (DOCX 20 KB) [file 11136_2019_2104_MOESM1_ESM.docx]

***Title:*** ASSOCIATION OF NUTRITIONAL STATUS AND HEALTH-RELATED QUALITY OF LIFE IN CHILDREN WITH CHRONIC KIDNEY DISEASE

***Short Title:*** HRQoL and nutritional status in paediatric CKD

***Authors:***

Dr Matthew Harmer BSc(Hons) BM MRCPCH ^a, b, d^,

Clinical Research Fellow in Paediatric Nephrology.

Dr Stephen Wootton BSc(Hons) PhD ^b, d^

Senior Lecturer in Human Nutrition.

Dr Rodney Gilbert MA, Mmed(Paed), FRCPCH, DCH ^a, b^

Consultant and Honorary Lecturer in Paediatric Nephrology.

Dr Caroline Anderson BSc(Hons), RD, PhD ^a, c, d^

Highly Specialist Paediatric Renal Dietitian.

Affiliations:

1. Southampton Children’s Hospital, University Hospital Southampton NHS Foundation Trust, Tremona Road, Southampton SO16 6YD, UK
2. University of Southampton, University Road, Southampton ,SO17 1BJ, UK
3. Department of Nutrition and Dietetics, University Hospital Southampton NHS Foundation Trust, Tremona Road, Southampton SO16 6YD, UK
4. NIHR Southampton Biomedical Research Centre–Nutrition, University Hospital Southampton NHS Foundation Trust, Southampton General Hospital, E level, Tremona Road, Southampton SO16 6YD, UK

***Corresponding Author:*** Dr Matthew Harmer: [matthew.harmer@uhs.nhs.uk](mailto:matthew.harmer@uhs.nhs.uk)

ORCID Number: 0000-0003-2977-0263

**Supplementary Material**

Correlation analysis between HRQoL scores and blood micronutrient levels.

|  | Child (Self-rater) PedsQL^TM^ | Parent-proxy PedsQL^TM^ |
| --- | --- | --- |
| Serum Copper | Spearman’s rho = -0.105,  p = 0.553. | Spearman’s rho = -0.255,  p = 0.104. |
| Serum Selenium | Spearman’s rho = -0.183,  p = 0.299. | Spearman’s rho = 0.165,  p = 0.295. |
| Serum Zinc | Spearman’s rho = -0.324,  p = 0.061. | Spearman’s rho = -0.180,  p = 0.254 |
| Whole Blood Manganese | Spearman’s rho = -0.260,  p = 0.173 | Spearman’s rho = -0.060,  p = 0.731. |

Mean Child (Self-rater) versus Parent-proxy PedsQLTM Scores of the cohort of paediatric pre-dialysis, conservatively-managed CKD patients, and test for child-parent concordance using paired t-test analysis.

|  |  | Physical domain | Emotional domain | Social domain | School domain | Total score |
| --- | --- | --- | --- | --- | --- | --- |
| 2 - 4 years  N=7 | Parent-proxy | 70.99 ± 27.88 | 61.43 ± 27.49 | 76.43 ±27.65 | 71.43 ± 26.29 | 68.76 ± 24.31 |
| 5 – 7 years  N=8 | Child(self-rater) | 54.02 ± 31.13 | 61.88 ± 32.06 | 55.73 ± 30.41 | 53.75 ± 16.85 | 56.01 ± 24.18 |
|  | Parent-proxy | 52.12 ± 28.54 | 47.50 ± 17.53 | 67.50 ± 24.35 | 55.00 ±23.30 | 55.02 ± 20.16 |
|  | Paired t-test Result | T(7) = 0.329,  P = 0.752 | T(7) = 2.308  P = 0.054 | T(7) = -1.555,  P = 0.164 | T(7 ) =-0.166,  P = 0.873 | T(7) = 0.244,  P = 0.814 |
| 8 – 12 years  N=15 | Child(self-rater) | 70.24 ± 19.10 | 66.33 ± 17.16 | 67.33 ± 18.70 | 62.83 ±16.44 | 67.23 ± 14.92 |
|  | Parent-proxy | 73.18 ±26.64 | 63.67 ± 24.31 | 68.33 ± 22.73 | 64.33 ±15.22 | 67.33 ± 18.37 |
|  | Paired t-test Result | T(12) = -0.467,  P = 0.649 | T(12) = 0.600,  P = 0.560 | T(12) = -0.457,  P = 0.656 | T(12) = -0.241,  P = 0.814 | T(12 ) =0.044,  P = 0.966 |
| 13 – 18 years  N=16 | Child(self-rater) | 70.24 ± 19.10 | 66.33 ± 17.16 | 67.33 ±18.70 | 62.83 ±16.44 | 67.23 ± 14.92 |
|  | Parent-proxy | 73.18 ± 26.64 | 63.67 ± 24.31 | 68.33 ± 22.73 | 64.33 ± 15.22 | 67.33 ± 18.37 |
|  | Paired t-test Result | T(14) = 0.846,  P = 0.412 | T(14 ) =-0.108,  P = 0.915 | T(14) = 0-.343,  P = 0.737 | T(14) = -1079,  P = 0.299 | T(14) = 0.355,  P=0.728 |
| Total  N=46 | Child(self-rater) | 65.10 ± 22.57 | 64.38 ± 21.65 | 65.48 ± 23.98 | 58.88 ± 16.91 | 64.22 ± 18.00 |
|  | Parent-proxy | 65.63 ± 40.63*†* | 61.47 ± 22.53 | 75.00 ± 35.00*†* | 63.48 ± 19.68 | 64.64 ± 19.13 |
|  | Paired t-test Result | T(37) = 0.179,  P = 0.859 | T(37) = 1.249,  P = 0.220 | T(37 ) = -1.045,  P = 0.303 | T(37) = -1.056,  P = 0.298 | T(37) = 0.281,  P = 0.780 |

*Scores expressed as percentages with standard deviation in parentheses.† - median and interquartile range.. Nb. there is no self-reported score for those aged 2-4years. P-values refer to difference between the self-reported and parent-proxy score for each age-group.*
